# Absence of *Mycoplasma genitalium* Strains Simultaneously Harboring Wild-Type *gyrA*, *parC*, and 23S rRNA Genes in Japan: Next-Generation Sequencing–Based Surveillance

**DOI:** 10.1093/ofid/ofag510

**Published:** 2026-08-12

**Authors:** Ayane Nishino, Natsumi Haba, Seiya Sawada, Kosuke Mizutani, Koji Kameyama, Shingo Kamei, Shigeaki Yokoi, Satoshi Ishihara, Masaki Takanashi, Yoshihisa Ashihara, Yuji Sekiguchi, Shin Ito, Mitsuru Yasuda, Takashi Deguchi

**Affiliations:** Cancer Genomic Testing and Treatment Center, Central Japan International Medical Center, Minokamo, Gifu, Japan; Cancer Genomic Testing and Treatment Center, Central Japan International Medical Center, Minokamo, Gifu, Japan; Cancer Genomic Testing and Treatment Center, Central Japan International Medical Center, Minokamo, Gifu, Japan; Department of Urology, Central Japan International Medical Center, Minokamo, Gifu, Japan; Department of Urology, Central Japan International Medical Center, Minokamo, Gifu, Japan; Department of Urology, Central Japan International Medical Center, Minokamo, Gifu, Japan; Department of Urology, Central Japan International Medical Center, Minokamo, Gifu, Japan; Department of Urology, Central Japan International Medical Center, Minokamo, Gifu, Japan; Infectious Disease Testing Department, LSI Medience Corporation, Tokyo, Japan; Clinical Laboratory, Medical Corporation Yoboukai, Tokyo, Japan; Clinical Laboratory, Medical Corporation Yoboukai, Tokyo, Japan; iClinic, Sendai, Miyagi, Japan; Department of Infection Control and Laboratory Medicine, Sapporo Medical University, Sapporo, Hokkaido, Japan; Cancer Genomic Testing and Treatment Center, Central Japan International Medical Center, Minokamo, Gifu, Japan

**Keywords:** 23S rRNA, *gyrA*, *Mycoplasma genitalium*, next-generation sequencing, *parC*

## Abstract

Using next-generation sequencing, we analyzed fluoroquinolone and macrolide resistance-associated mutations of the *gyrA*, *parC*, and 23S rRNA genes in *Mycoplasma genitalium* and detected mutations in 22.3% (37/166), 92.5% (161/174), and 96.0% (169/176), respectively, in Japan. No strains harboring 3 wild-type genes were identified, indicating widespread dissemination of antimicrobial resistance.

*Mycoplasma genitalium* is a recognized causative pathogen of sexually transmitted infections. Tetracycline-class antibiotics have shown limited efficacy for the treatment of *M. genitalium* infections [1]. Although macrolide regimens were previously recommended, their clinical effectiveness has declined because of the emergence and spread of *M. genitalium* strains harboring macrolide resistance-associated mutations in the 23S rRNA gene [2]. Mutations in domain V of the 23S rRNA gene reduce macrolide binding to the bacterial ribosome, resulting in decreased susceptibility to macrolides.

For persistent *M. genitalium* infections after macrolide treatment, moxifloxacin has been used worldwide, and sitafloxacin has also been recommended in Japan [3]. However, treatment failures associated with fluoroquinolone-resistant *M. genitalium* have been increasingly reported [4]. Fluoroquinolones target both DNA gyrase and topoisomerase IV. The *gyrA* and *parC* genes encode the GyrA and ParC subunits of these enzymes, respectively. In *M. genitalium*, amino acid substitutions in the quinolone resistance-determining region of *parC* are the primary determinants of fluoroquinolone resistance, and additional *gyrA* mutations may further decrease fluoroquinolone susceptibility by reducing fluoroquinolone binding to their enzyme–DNA complexes. Concurrent mutations in *parC* and *gyrA* have been associated with higher fluoroquinolone minimum inhibitory concentrations [5] and treatment failure [4, 6].

Antimicrobial resistance surveillance of *M. genitalium* has been conducted primarily by detecting genetic alterations associated with resistance due to the organism's fastidious nature and difficult isolation from clinical specimens, rendering phenotypic antimicrobial susceptibility testing impractical. We previously performed macrolide and fluoroquinolone resistance surveillance of *M. genitalium* by analyzing the 23S rRNA, *gyrA*, and *parC* genes using PCR amplification followed by Sanger sequencing [7]. This approach required substantial labor, time, and cost when applied to a large number of clinical specimens.

In the present study, we employed next-generation sequencing (NGS) to analyze mutations within the 23S rRNA, *gyrA*, and *parC* genes of *M. genitalium*. We aimed to evaluate the utility of NGS-based genetic analysis as a practical tool for antimicrobial resistance surveillance and to clarify the current status of antimicrobial resistance in *M. genitalium* in Japan.

## METHODS

We analyzed *M. genitalium*–positive specimens across Japan during February–August 2025, following approval from the Institutional Review Board of Central Japan International Medical Center (Minokamo, Japan). Residual clinical specimens that were positive for *M. genitalium* by the cobas® TV/MG assay (Roche Diagnostics, Basel, Switzerland) were collected without selection according to individual clinical information from 6 clinics in Tokyo and 1 clinic in each of Kawasaki, Yokohama, Nagoya, Osaka, and Fukuoka in Japan (Supplementary Figure 1). One specimen from each patient was included in this study. We analyzed 178 specimens (64 male urine and 114 female cervical swab samples). Geographically, the specimens were distributed as follows: 106 (59.6%) from Tokyo, 18 (10.1%) from Kawasaki, 17 (9.6%) from Yokohama, 9 (5.1%) from Nagoya, 12 (6.7%) from Osaka, and 16 (9.0%) from Fukuoka. Testing for *M. genitalium* has been recommended only for symptomatic patients with non-gonococcal urethritis or cervicitis in Japan since it was available in clinical setting in 2022 [3]. All specimens were fully anonymized and not linked to any identifiable clinical data. Therefore, no information regarding symptom status or indication for *M. genitalium* testing was available for each specimen. Informed consent was not obtained from the patients.

Target regions of the 23S rRNA, *gyrA*, and *parC* genes were amplified using in-house multiplex PCR with the primer sets listed in Supplementary Tables 1 and 2 and analyzed by NGS on an Illumina MiSeq platform. Detailed protocols are described in the Supplementary Methods. For specimens with incomplete NGS results, Sanger sequencing was performed [7].

Differences in prevalence of individual mutations and variant patterns between sexes and among cities were analyzed using Fisher's exact test or Pearson's χ² test, as appropriate. A *P* value of <.05 was considered to indicate statistical significance.

## RESULTS

Next-generation sequencing successfully determined nucleotide sequences in 91.6% (163/178) of specimens for *gyrA*, 94.4% (168/178) for *parC*, and 94.9% (169/178) for 23S rRNA. After supplementation with Sanger sequencing, final analyses included 166, 174, and 176 specimens, respectively. Genotyping of all 3 genes was achieved in 164 specimens, which were included in the resistance pattern analysis.

Five *gyrA* mutations resulting in GyrA amino acid substitutions (Met95Ile, Met95Val, Asp99Asn, Asp99Gly, and Asp99Tyr) were detected in 22.3% (37/166) of specimens, most commonly an amino acid substitution of Met95Ile, whereas 6 *parC* mutations resulting in ParC amino acid substitutions (Ser83Arg, Ser83Asn, Ser83Ile, Asp87Asn, Asp87His, Asp87Tyr) were observed in 92.5% (161/174), predominantly Ser83Ile (62.6%) (Table 1). Among the amino acid substitutions, Met95Ile, Asp99Asn, and Asp99Tyr in GyrA, and Ser83Ile and Asp87Tyr in ParC, have been demonstrated to be associated with reduced susceptibility to fluoroquinolones [5, 8]. Conversely, the association between reduced fluoroquinolone susceptibility and the other amino acid substitutions at the same positions in GyrA and ParC remains unestablished. Amino acid residues 95 and 99 of *M. genitalium* GyrA correspond to residues 83 and 87 of *Escherichia coli* GyrA, respectively. Likewise, residues 83 and 87 of *M. genitalium* ParC correspond to residues 80 and 84 of *E. coli* ParC, respectively. In a variety of bacterial species, amino acid substitutions at the positions corresponding to GyrA residues 83 and 87 and ParC residues 80 and 84 of *E. coli* are well recognized to be associated with reduced susceptibility to fluoroquinolones. Therefore, for the purposes of this molecular surveillance study, we defined genetic mutations resulting in amino acid substitutions at residues 95 and 99 of GyrA and at residues 83 and 87 of ParC as fluoroquinolone resistance-associated mutations.

**Table 1.** Prevalence of Variants in the *gyrA*, *parC*, and 23S rRNA Genes in Clinical Specimens

Gene/Variant	All, *n* (%)	Male, *n* (%)	Female, *n* (%)	*P* Value^a^	Tokyo, *n* (%)	Yokohama, *n* (%)	Kawasaki, *n* (%)	Nagoya, *n* (%)	Osaka, *n* (%)	Fukuoka, *n* (%)	*P* Value^b^
*gyrA*/wild	129 (77.7)	49 (84.5)	80 (74.1)	0.171	73 (76.8)	10 (58.8)	16 (94.1)	8 (88.9)	8 (66.7)	14 (87.5)	0.128
*gyrA*/Met95Ile^c^	15 (9.0)	3 (5.2)	12 (11.1)	0.263	13 (13.7)	1 (5.9)	0 (0.0)	0 (0.0)	1 (8.3)	0 (0.0)	0.230
*gyrA*/Met95Val	3 (1.8)	1 (1.7)	2 (1.9)	1.000	0 (0.0)	0 (0.0)	0 (0.0)	1 (11.1)	2 (16.7)	0 (0.0)	0.002
*gyrA*/Asp99Asn^c^	11 (6.6)	5 (8.6)	6 (5.6)	0.518	7 (7.4)	1 (5.9)	0 (0.0)	0 (0.0)	1 (8.3)	2 (12.5)	0.716
*gyrA*/Asp99Gly	3 (1.8)	0 (0.0)	3 (2.8)	0.552	1 (1.1)	2 (11.8)	0 (0.0)	0 (0.0)	0 (0.0)	0 (0.0)	0.055
*gyrA*/Asp99Tyr^c^	5 (3.0)	0 (0.0)	5 (4.6)	0.164	1 (1.1)	3 (17.6)	1 (5.9)	0 (0.0)	0 (0.0)	0 (0.0)	0.009
*parC*/wild	13 (7.5)	6 (10.0)	7 (6.1)	0.375	9 (8.7)	0 (0.0)	2 (12.5)	2 (22.2)	0 (0.0)	0 (0.0)	0.202
*parC/*Ser83Arg	2 (1.1)	2 (3.3)	0 (0.0)	0.118	1 (1.0)	0 (0.0)	0 (0.0)	0 (0.0)	1 (8.3)	0 (0.0)	0.291
*parC*/Ser83Asn	33 (19.0)	18 (30.0)	15 (13.2)	0.014	15 (14.4)	0 (0.0)	6 (37.5)	2 (22.2)	4 (33.3)	6 (37.5)	0.014
*parC*/Ser83Ile^c^	109 (62.6)	27 (45.0)	82 (71.9)	0.001	68 (65.4)	14 (82.4)	7 (43.8)	5 (55.6)	7 (58.3)	8 (50.0)	0.222
*parC*/Asp87Asn	7 (4.0)	2 (3.3)	5 (4.4)	1.000	6 (5.8)	1 (5.9)	0 (0.0)	0 (0.0)	0 (0.0)	0 (0.0)	0.670
*parC*/Asp87His	1 (0.6)	0 (0.0)	1 (0.9)	1.000	0 (0.0)	0 (0.0)	0 (0.0)	0 (0.0)	0 (0.0)	1 (6.3)	0.077
*parC*/Asp87Tyr^c^	9 (5.2)	5 (8.3)	4 (3.5)	0.278	5 (4.8)	2 (11.8)	1 (6.3)	0 (0.0)	0 (0.0)	1 (6.3)	0.738
23S rRNA/wild	6 (3.4)	3 (4.8)	3 (2.6)	0.427	6 (5.8)	0 (0.0)	0 (0.0)	0 (0.0)	0 (0.0)	0 (0.0)	0.507
23S rRNA/A2071G^d^	41 (23.3)	21 (33.9)	20 (17.5)	0.024	21 (20.2)	0 (0.0)	5 (27.8)	4 (44.4)	5 (41.7)	6 (37.5)	0.031
23S rRNA/A2071T	16 (9.1)	7 (11.3)	9 (7.9)	0.584	10 (9.6)	3 (17.6)	2 (11.1)	0 (0.0)	0 (0.0)	1 (6.3)	0.566
23S rRNA/A2072G^d^	112 (63.6)	31 (50.0)	81 (71.1)	0.001	67 (64.4)	14 (82.4)	11 (61.1)	5 (55.6)	6 (50.0)	9 (56.3)	0.515
23S rRNA/A2073G	1 (0.6)	0 (0.0)	1 (0.9)	1.000	0 (0.0)	0 (0.0)	0 (0.0)	0 (0.0)	1 (8.3)	0 (0.0)	0.017

^a^Differences in the prevalence of individual variants in the *gyrA*, *parC*, and 23S rRNA genes between sexes were analyzed using Fisher's exact test.

^b^Differences in the prevalence of individual variants in the *gyrA*, *parC*, and 23S rRNA genes among cities were analyzed using Pearson's χ² test.

^c^Among the amino acid substitutions, Met95Ile, Asp99Asn, and Asp99Tyr in GyrA, and Ser83Ile and Asp87Tyr in ParC, have been demonstrated to be associated with reduced susceptibility to fluoroquinolones [5, 8].

^d^Mutations in the 23S rRNA gene, A2071G and A2072G, have been demonstrated to be associated with reduced susceptibility to macrolides [5].

Four mutations in the 23S rRNA gene (A2071G, A2071T, A2072G, A2073G) were identified. Mutations at A2071 and A2072 have been reported to reduce susceptibility to macrolides [5]. Therefore, A2071G, A2071T, and A2072G were defined as macrolide resistance-associated mutations in this study. These mutations were detected in 96.0% (169/176) of specimens, with A2072G being the most prevalent variant.

Statistically significant differences were observed between male and female patients in some individual mutations of the *parC* and 23S rRNA genes, and regional variation was present for some mutations in all *gyrA*, *parC*, and 23S rRNA genes.

Among the 164 specimens with genotyping of all 3 genes, 37 (22.6%) harbored *gyrA* mutations, and all exhibited concomitant mutations in both the *parC* and 23S rRNA genes (Table 2). The number of strains with *parC* mutations in the absence of *gyrA* mutations was 114 (69.5%). The most common resistance pattern was mutant *parC* and 23S rRNA with wild-type *gyrA* and observed in 110 (67.1%). The prevalence of strains harboring both fluoroquinolone- and macrolide-associated mutations was 89.6% (147/164). Notably, no strains simultaneously harboring wild-type *gyrA*, *parC*, and 23S rRNA genes were identified.

**Table 2.** Prevalence of Variant Patterns of the *gyrA*, *parC*, and 23S rRNA Genes in Clinical Specimens

Resistance-Associated Mutation In			All, *n* (%)	Male, *n* (%)	Female, *n* (%)	*P* Value^a^	Tokyo, *n* (%)	Kawasaki, *n* (%)	Yokohama, *n* (%)	Nagoya, *n* (%)	Osaka, *n* (%)	Fukuoka, *n* (%)	*P* Value^b^
gyrA	parC	23S rRNA											
Present	Present	Present	37 (22.6)	9 (16.1)	28 (25.9)	0.177	22 (23.4)	1 (6.3)	7 (41.2)	1 (11.1)	4 (33.3)	2 (12.5)	0.175
Absent	Present	Present	110 (67.1)	40 (71.4)	70 (64.8)	0.485	60 (63.8)	13 (81.3)	10 (58.8)	6 (66.7)	7 (58.3)	14 (87.5)	0.355
Absent	Present	Absent	4 (2.4)	1 (1.8)	3 (2.8)	1.000	3 (3.2)	0 (0.0)	0 (0.0)	0 (0.0)	1 (8.3)	0 (0.0)	0.584
Absent	Absent	Present	13 (7.9)	6 (10.7)	7 (6.5)	0.380	9 (9.6)	2 (12.5)	0 (0.0)	2 (22.2)	0 (0.0)	0 (0.0)	0.198
Absent	Absent	Absent	0 (0.0)	0 (0.0)	0 (0.0)	NA	0 (0.0)	0 (0.0)	0 (0.0)	0 (0.0)	0 (0.0)	0 (0.0)	NA^c^

^a^Differences in the prevalence of individual variant patterns of the *gyrA*, *parC*, and 23S rRNA genes between sexes were analyzed using Fisher's exact test.

^b^Differences in the prevalence of individual variant patterns of the *gyrA*, *parC*, and 23S rRNA genes among cities were analyzed using Pearson's χ² test.

^c^Not applicable.

No statistically significant differences in individual resistance pattern frequencies were observed between male and female patients or among cities (Table 2). Distributions of resistance patterns showed no statistically significant differences between sexes (*P* = .458) or among cities (*P* = .318).

## DISCUSSION

In this study, NGS enabled high-throughput and comprehensive detection of resistance-associated mutations, with detection rates exceeding 90% for all target genes. Incomplete NGS results for a small fraction of specimens could be attributed to low bacterial DNA loads and/or DNA degradation in clinical specimens. For large-scale analyses, such as surveillance studies, NGS offers clear advantages over time- and labor-intensive Sanger sequencing. Moreover, scalable custom amplicon sequencing based on NGS for multiple target genes could offer a more cost-effective alternative to Sanger sequencing [9].

In this study, mutation proportions were 96.0%, 22.6%, and 92.5% in the 23S rRNA, *gyrA*, and *parC* genes, respectively. The prevalence of strains harboring both macrolide and fluoroquinolone resistance-associated mutations was 89.6%. A recent meta-analysis, which included strains of *M. genitalium* between 2018 and 2021, estimated global prevalence rates of macrolide resistance-associated mutations at 33.3%, fluoroquinolone resistance-associated mutations at 13.3%, and dual-class antimicrobial resistance-associated mutations at 6.5% [10]. More recent surveillance data on specimens collected in 2024 reported the prevalence of macrolide resistance-associated mutations, fluoroquinolone resistance-associated mutations, and dual macrolide and fluoroquinolone resistance-associated mutations of 63.6%, 16.7%, and 13.7%, respectively, in the United Kingdom [11], whereas the corresponding prevalences in Korea were 31.2%, 32.5%, and 19.4% [12]. Although our study analyzed more recently collected specimens, it demonstrated an exceptionally high prevalence of antimicrobial resistance-associated mutations in Japan.

Our previous nationwide surveillance analyzed specimens collected between 2013 and 2017 in Japan [7]. The prevalence of mutations in the 23S rRNA gene increased over time from approximately 40% in 2013 to over 70% in 2017, whereas those in the *gyrA* and *parC* genes increased from approximately 3% to 10% in 2017 and 50% to 70% in 2017, respectively. The prevalence of strains harboring both macrolide and fluoroquinolone resistance-associated mutations reached approximately 50% in 2016 and 2017. Contrastingly, the prevalence of strains lacking mutations in all 3 genes decreased over time, falling below 20% in 2016 and 2017. Subsequent studies in Japan have shown further increases in the prevalence of mutations in the 23S rRNA, *gyrA*, and *parC* genes [13].

In this study, no strains simultaneously harboring wild-type *gyrA*, *parC*, and 23S rRNA genes were identified. Mutations in all the genes were consistently detected in strains from all participating cities. No statistically significant differences were observed in resistance patterns between male and female patients and among cities. These findings provide robust evidence that antimicrobial-resistant *M. genitalium* strains are widely disseminated throughout Japan and that resistance has reached an advanced and potentially saturated stage at the population level.

In Japan, fluoroquinolones with activity against *Chlamydia trachomatis*, such as ofloxacin, tosufloxacin, levofloxacin, and gatifloxacin, were developed and introduced into clinical practice for the treatment of non-gonococcal urethritis and cervicitis between the mid-1980s and early 2000s. As early as 2001, we detected fluoroquinolone resistance-associated mutations in the *parC* gene of *M. genitalium* from men with non-gonococcal urethritis who had been treated with levofloxacin [14]. The widespread clinical use of fluoroquinolones may therefore have contributed to the selection of fluoroquinolone-resistant *M. genitalium* strains in Japan.

The approved regimen of azithromycin for the treatment of sexually transmitted infections is limited to a single 1-g dose in Japan, though this regimen is no longer recommended as first-line treatment for non-gonococcal urethritis [3]. The lack of alternative dosing strategies, such as extended regimens, may have contributed to the selection and persistence of macrolide-resistant strains.

Widespread dissemination of resistant strains has critical clinical implications. The extremely high prevalence of 23S rRNA macrolide resistance-associated mutations (96.0%) suggests that macrolide-based therapy is no longer effective as empirical treatment for non-gonococcal urethritis and cervicitis. The prevalence of strains simultaneously harboring mutations in both *gyrA* and *parC* was 22.3% in this study. Such strains are considered highly resistant [7] and unlikely to respond to sitafloxacin or moxifloxacin [4, 6]. Although strains with *parC* fluoroquinolone resistance-associated mutations alone may retain partial susceptibility [5], their high prevalence (69.5%) raises concern that these strains may serve as a reservoir for the subsequent acquisition of *gyrA* mutations. Given the extremely low prevalence of antimicrobial-susceptible strains as demonstrated in this study, resistance-guided therapy may have limited utility due to the scarcity of effective treatment options. Therefore, alternative practical interim strategies are urgently needed to achieve better clinical outcomes and minimize further selection of resistance. These include combination, sequential, and/or prolonged regimens until new antimicrobial agents with activity against antimicrobial-resistant *M. genitalium* become available [15] and withholding *M. genitalium* screening and antimicrobial treatment for asymptomatic individuals [3].

We are fully aware of several limitations in this study. The sample size was relatively small. The number of clinical specimens differed across cities. Moreover, detailed individual clinical data, including symptom status, indication for testing of *M. genitalium*, prior antimicrobial exposure, and treatment outcomes, were not available. Therefore, statistically significant differences in mutation frequencies observed in this study should be interpreted with caution.

In conclusion, this study suggested that NGS would be a useful and practical approach for large-scale surveillance involving multiple gene targets. This study showed the near-complete absence of fully susceptible *M. genitalium* strains in Japan as no strains simultaneously harboring wild-type *gyrA*, *parC*, and 23S rRNA genes were identified. Despite this study's limitations, it provides important insights into the current resistance status of *M. genitalium* in Japan, underscoring the urgent need to revise current treatment strategies and develop new therapeutic options.

## Supplementary Material

ofag510_Supplementary_Data

## Supplementary Data

Supplementary materials are available at *Open Forum Infectious Diseases* online. Consisting of data provided by the authors to benefit the reader, the posted materials are not copyedited and are the sole responsibility of the authors, so questions or comments should be addressed to the corresponding author.
