# Supplementary material for: Absence of Mycoplasma genitalium Strains Simultaneously Harboring Wild-Type gyrA, parC, and 23S rRNA Genes in Japan: Next-Generation Sequencing–Based Surveillance
Source: Open Forum Infect Dis. 2026 Aug 12;13(8):ofag510. doi: 10.1093/ofid/ofag510 (PMC13507314; doi:10.1093/ofid/ofag510)
Supplement: Supplementary file 1 [file ofag510_supplementary_data.docx]

**Supplementary Figure.** Geographic locations of the cities where clinical specimens were collected, shown on the map of Japan.

**Supplementary Methods**

**Collection of clinical specimens and DNA purification**

Urine specimens were collected using the Aptima® Urine Specimen Collection Kit (Hologic, Inc., Marlborough, MA, USA), and swab specimens were collected using the Aptima® Unisex Swab Specimen Collection Kit (Hologic, Inc., Marlborough, MA, USA), in accordance with the manufacturer’s instructions. DNA was extracted automatically on the QIAsymphony® SP instrument (QIAGEN, Hilden, Germany) using the QIAsymphony® DSP Virus/Pathogen Kit (QIAGEN, Hilden, Germany), according to the manufacturer’s protocol.

**PCR amplification and library preparation**

PCR amplification of the three target loci was performed using primer sets listed in Supplementary Tables 1 and 2. A three-step PCR protocol was employed, and all PCR reactions were performed as multiplex PCRs.

In the first PCR step, one primer set per gene region was mixed to simultaneously amplify the three target regions. In the second PCR step, the amplicons generated in the first step were used as templates and amplified using tailed primers containing universal adapter sequences at the 5′ ends of the gene-specific primers (tailed PCR method). This step enabled the attachment of sequencing adapters in the subsequent index PCR.

The first and second PCR reactions were performed in a total volume of 26.7 µL, consisting of 10 µL DNase-free water, 12.5 µL 2× KAPA HiFi HotStart ReadyMix, 1.67 µL primer mix (each primer at a final concentration of 0.07 µM), and 2.5 µL template DNA. The cycling conditions for the first PCR were as follows: initial denaturation at 95°C for 3 min; 40 cycles of denaturation at 95°C for 30 s, annealing at 63°C for 30 s, and extension at 72°C for 30 s; followed by a final extension at 72°C for 5 min and a hold at 4°C.

The cycling conditions for the second PCR were as follows: initial denaturation at 95°C for 3 min; 40 cycles of denaturation at 95°C for 30 s, annealing at 61.1°C for 30 s, and extension at 72°C for 30 s; followed by a final extension at 72°C for 5 min and a hold at 4°C. The universal tail sequences were as follows: forward universal tail, 5′-TCGTCGGCAGCGTCAGATGTGTATAAGAGACAG-[gene-specific sequence]; reverse universal tail, 5′-GTCTCGTGGGCTCGGAGATGTGTATAAGAGACAG-[gene-specific sequence].

In the third PCR step, index and adapter sequences were added using the IDT for Illumina DNA/RNA UD Index Sets A–C (Illumina, San Diego, CA, USA), with a unique index assigned to each sample. The third PCR reaction was performed in a total volume of 50 µL, consisting of 10 µL DNase-free water, 25 µL 2× KAPA HiFi HotStart ReadyMix, 10 µL index primer mix, and 5.0 µL template DNA. Cycling conditions were as follows: initial denaturation at 95°C for 3 min; 8 cycles of denaturation at 95°C for 30 s, annealing at 55°C for 30 s, and extension at 72°C for 30 s; followed by a final extension at 72°C for 5 min and a hold at 4°C.

**Gel electrophoresis and library quantification**

PCR amplicon size and quality were evaluated using a TapeStation 4200 system (Agilent Technologies, Santa Clara, CA, USA) with the D1000 Reagent Kit, according to the manufacturer’s instructions. Expected amplicon size ranges are summarized in Supplementary Table 1. Because nested PCR efficiently enriched the target amplicons, purification of PCR products was omitted.

Library concentrations were calculated based on TapeStation results using the following formula:

Library concentration (nM) =
[concentration (ng/µL) / (660 g/mol × average library size)] × 10^6^,

where 660 g/mol represents the average molecular weight per base pair of double-stranded DNA.

Libraries were pooled to a final concentration of 16 nM. The pooled library was quantified fluorometrically using the Qubit 1× dsDNA HS Assay Kit (Thermo Fisher Scientific, Waltham, MA, USA), and the library concentration was recalculated using the same formula. The pooled library was then adjusted to 8 pM and denatured at 96°C for 2 min.

**Next-generation sequencing**

A 600 µL aliquot of the denatured library pool, including a 25% PhiX control spike-in, was loaded onto MiSeq Reagent Nano Kit v2 (300 cycles) cartridges and sequenced using a MiSeq instrument (Illumina). Three target regions were multiplexed for each sample, and sufficient sequencing depth was achieved to ensure a minimum coverage of approximately 100× per target region.

**Bioinformatic analysis**

Primary data processing, including base calling and conversion of signal intensities to BCL files, was performed using Real-Time Analysis (RTA; Illumina). Secondary analysis was conducted using Local Run Manager (Illumina) with the Resequencing Module to generate FASTQ and BAM files.

Sequencing reads were aligned to the reference genome NC_000908.2 (*Mycoplasma genitalium* G37, complete sequence). Variant calling was performed using the Genome Analysis Toolkit (GATK). Variants with a variant allele frequency (VAF) ≥1.0% were considered true mutations, whereas variants with a VAF <1.0% were excluded because they could not be reliably distinguished from sequencing errors. Only reads with lengths consistent with the expected amplicon sizes listed in Supplementary Table 2 were included in the analysis.

BAM files were further inspected using GenomeJack software (Mitsubishi Space Software Co., Ltd., Tokyo, Japan), in which coverage depth and variant depth were examined and VAF (%) was calculated. Regions with insufficient read coverage were reported as “not detected.”

| **Supplementary Table 1.** Primers for the first PCR | | | | |
| --- | --- | --- | --- | --- |
| Locus | Primer for 1st PCR | Genomic position (G37-type strain) | Gene-specific sequence (5′＞3′) | PCR Amplicon (bp) |
| 23S rRNA | 23S-outer F | 173626 to 173644 | CTGAAGCCCCAGTGAACGG | 359 |
|  | 23S-outer R | 173964 to 173984 | ACTGCCCACCTAACACTGTCT |  |
| gyrA | GYRA-outer F | 4982 to 5003 | TCGTCGTGTTCTTTATGGTGCT | 231 |
|  | GYRA-R | 5193 to 5213 | ATAACGTTGTGCAGCAGGTCT |  |
| parC | PARC-outer F | 242373 to 242393 | TGGGATGTTTCAAATGGGCTT | 205 |
|  | PARC-outer R | 242558 to 242577 | TGCAGCATTATCCCCATCCA |  |

| **Supplementary Table 2.** Primers for the second PCR | | | | |
| --- | --- | --- | --- | --- |
| Locus | Primer for 2nd PCR | Genomic position (G37-type strain) | Gene-specific sequence (5′＞3′) | PCR Amplicon (bp) |
| 23S rRNA | 23S-F | 173697 to 173716 | CCGTCCCGCTTGAATGGTGT | 240 173+overhang |
|  | 23S-R | 173845 to 173869 | TGCTCCTACCTATTCTCTACATGGT |  |
| gyrA | GYRA-F | 5045 to 5065 | TGCGAGGATTGTTGGTGATGT | 235 168+overhang |
|  | GYRA-R | 5193 to 5213 | ATAACGTTGTGCAGCAGGTCT |  |
| parC | PARC-F | 242390 to 242410 | GCTTAAAACCCACCACTCCCT | 245 178+overhang |
|  | PARC-R | 242547 to 242567 | TCCCCATCCACTGAACCATTG |  |
